# Survival outcomes of segmentectomy and lobectomy for early stage non-small cell lung cancer: a systematic review and meta-analysis

**DOI:** 10.1186/s13019-024-02832-6

**Published:** 2024-06-22

**Authors:** Tongxin Li, Wang He, Xiaolong Zhang, Yu Zhou, Dong Wang, Shengyuan Huang, Xiangyang Li, Yong Fu

**Affiliations:** Department of Cardiothoracic Surgery, Dianjiang People’s Hospital of Chongqing, Chongqing. No.116 Beijie, Guixi Street, Dianjiang County, Chongqing, 408300 China

**Keywords:** Lobectomy, Segmentectomy, NSCLC, Survival

## Abstract

**Background:**

The question of whether segmentectomy and lobectomy have similar survival outcomes for patients with early-stage non-small cell lung cancer (NSCLC) is a matter of debate.

**Methods:**

A cohort study and randomized controlled trial were included, comparing segmentectomy and lobectomy, by utilizing computerized access to the Pubmed, Web of Science, and Cochrane Library databases up until July 2022. The Cochrane Collaboration tool was used to evaluate the randomized controlled trials, while the Newcastle-Ottawa Scale (NOS) was used to evaluate the cohort studies. Sensitivity analyses were also carried out.

**Results:**

The analysis incorporated 17 literature studies, including one randomized controlled trial and 16 cohort studies, and was divided into a segmentectomy group (*n* = 2081) and a lobectomy group (*n* = 2395) based on the type of surgery the patient underwent. Each study was followed up from 27 months to 130.8 months after surgery. Over survival (OS): HR = 1.14, 95%CI(0.97,1.32), *P* = 0.10; disease-free survival (DFS): HR = 1.13, 95%CI(0.91,1.41), *P* = 0.27; recurrence-free survival (RFS): HR = 0.95, 95%CI(0.81,1.12), *P* = 0.54.

**Conclusion:**

The results of the study suggest that the survival outcomes of the segmentectomy group were not inferior to that of the lobectomy group. Segmentectomy should therefore be considered as a treatment option for early stage NSCLC.

**Supplementary Information:**

The online version contains supplementary material available at 10.1186/s13019-024-02832-6.

## Introduction

Lung cancer is a leading cause of cancer-related deaths globally [1]. With growing public awareness and rapid advancements in imaging technologies and devices, early-stage NSCLC is being detected more frequently [2]. Since 1995, lobectomy with lymph node dissection has been considered the gold standard for stage I NSCLC surgery [3] This decision was based on a randomized controlled study conducted by the Lung Cancer Study Group (LCSG), which reported that patients who underwent limited resection, including wedge resection and segmentectomy, had lower overall survival (OS) and higher recurrence rates compared to those who underwent lobectomy. However, one limitation of this trial was that wedge section and segmentectomy were not performed separately. The lower OS and higher recurrence rates in the limited resection group may have been related to the failure of wedge section to effectively remove the lymph nodes.

More recently, in April 2022, researchers published the results of a multicenter, randomized controlled, non-inferiority trial that compared segmentectomy and lobectomy in the treatment of early-stage NSCLC [4]. The results showed that the 5-year survival and 5-year recurrence rates were comparable between the segmentectomy and lobectomy groups, with the latter group retaining more lung function. As a result, segmentectomy is now recommended as a standard procedure for patients with small-peripheral NSCLC.

To further evaluate the survival outcomes of patients with stage I NSCLC who underwent segmentectomy or lobectomy, we conducted a meta-analysis of published studies. Our review analyzed the available data quantitatively and compared the survival rates of patients who underwent these two surgical procedures.

## Methods

### Eligibility criteria

Inclusion criteria were as follows: (1)Studies enrolled patients with stage I or early-stage NSCLC; (2) Segmentectomy and lobectomy should be compared in the included study. (3) Enrolment studies should be cohort studies or randomized controlled studies; (4) reporting at least one interest, including OS disease-free survival (DFS) or recurrence-free survival (RFS); (5) Both groups of patients included in the study needed to be larger than 20; (6) English literature only. Exclusion: (1) Articles published by the same author or with duplicate data (2) Data for the study were based on data from the National Cancer Database (NCDB) and the Surveillance, Epidemiology, and End Results [5] because of overlapping study populations.(3) Literatures not available in full. The results of this systematic review were registered on PROSPERO and are available on https://www.crd.york.ac.uk/prospero/display_record.php? ID=CRD42022355702.

### Search strategy

PubMed, Web of Science and Cochrane Library were independently searched by two researchers (Li Tongxin and He Wang). As an example, PubMed was used and free words were added to the subject words and Boolean logical operators: (‘Carcinoma, Non-Small-Cell Lung’ [MESH terms]) and (‘Pneumonectomy’ [MESH terms] and (‘segmentectomy’ or ‘segmental resection’) and survival). A comprehensive search of literature was conducted up to July 2022 with no restrictions on the study design or publication status, whether published or unpublished. As a result, 549 related studies were retrieved from PubMed.”

### Data extraction

The data were independently extracted and cross-checked by two researchers (Li Tongxin and Zhang Xiaolong). Differences, if any, were discussed and resolved or decided by the third researcher. Data should be extracted from: first author, time of publication, author nationality, type of study, use of propensity score matching, study time, clinical stage, sample size, follow-up time, OS, DFS, RFS, HR and 95% CI obtained through Kaplan-Meier survival curve. Regarding the consistency between the 7th and 8th edition criteria for stage I lung cancer, we ensured alignment by carefully examining the literature and cross-referencing the tumor size thresholds.

### Quality assessment

The Cochrane collaboration tool was used by two authors (Li Tongxin and He Wang) to evaluate the quality of randomized controlled trials. Each study was evaluated from the following aspects: random sequences generation, allocation concealment, blinding of participants and personnel, blinding of outcome assessment, incomplete outcome data, selective reporting and other bias. Each bias was judged to be unclear, low or high risk. The retrospective cohort study was assessed using the Newcastle-Ottawa scale (NOS), which is widely used in non-randomized studies, including quality of selection, comparability and outcome of study participants to assess the quality of the study, with a full score of 9, with a total score of 8 or 9 as high quality and a total score of 6 or 7 as medium quality. If differences arose, they were resolved through discussion among researchers.

### Statistical analysis

Data extracted from the literature were statistically analyzed using the Review Manager 5.4 software(The Cochrane Collaboration Oxford, UK). The hazard ratio (HR) and its standard error (SE) were used to analyze the survival data (OS, DFS and RFS). If HR is not directly reported in the included study, Kaplan-Meier survival curve data were extracted to calculate HR based on Tierney et al [6]. The Kaplan-Meier survival curve was read by the Engauge Digitizer software. (The software is free and available to download from https://sourceforge.net/projects/digitizer/files/). All the calculations were done independently by two researchers(Li Tongxin and He wang)and if there were differences, they were resolved through discussion. Heterogeneity included in the study was assessed using I^2^ statistics [7], and if the heterogeneity was not significant (I^2^ ≤ 50, *P* > 0.1), the fixed effect model was used for combined analysis, otherwise the random effect model was used for analysis. We use funnel plots to assess publication bias. Prespecified sensitivity analyses were performed to evaluate the influence of each study by excluding each study one by one. The data of the study using the propensity score matching method were analyzed separately.

## Results

### Search results

**Fig. 1:**
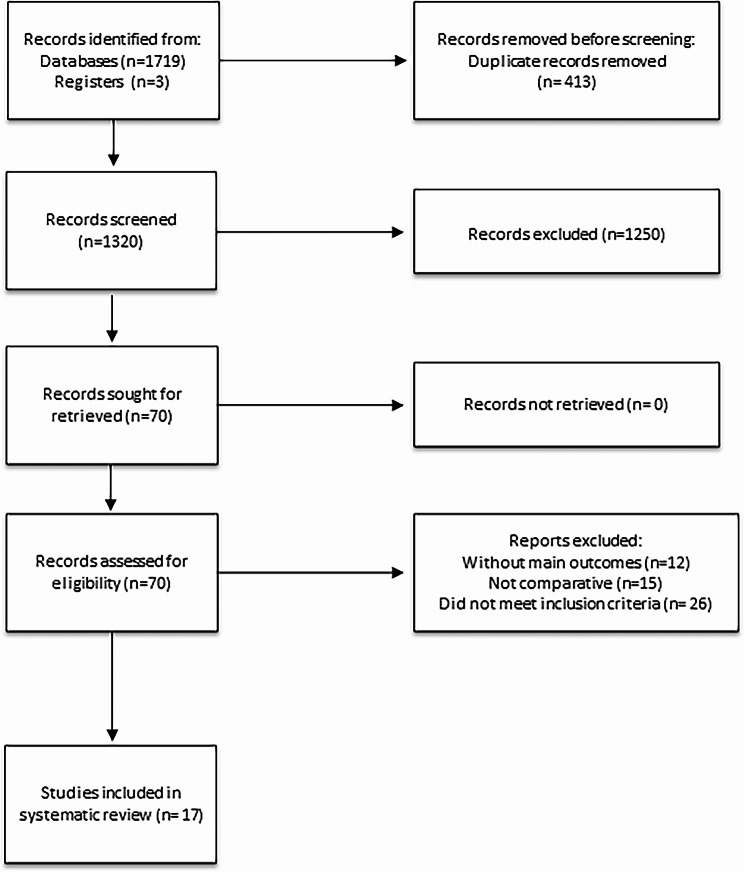
The PRISMA 2020 flowchart of the selection process to identify studies eligible for pooling

The manual search of reference lists and the electronic database search yielded a total of 1722 publications. Among them, 17 retrospective studies met our eligibility criteria [5]. (as depicted in Fig. 1). To prevent duplication of patient data, the meta-analysis excluded studies based on the Surveillance, Epidemiology and End Results [5] database and the National Cancer Database (NCDB).

### Quality assessment

**Fig. 2:**
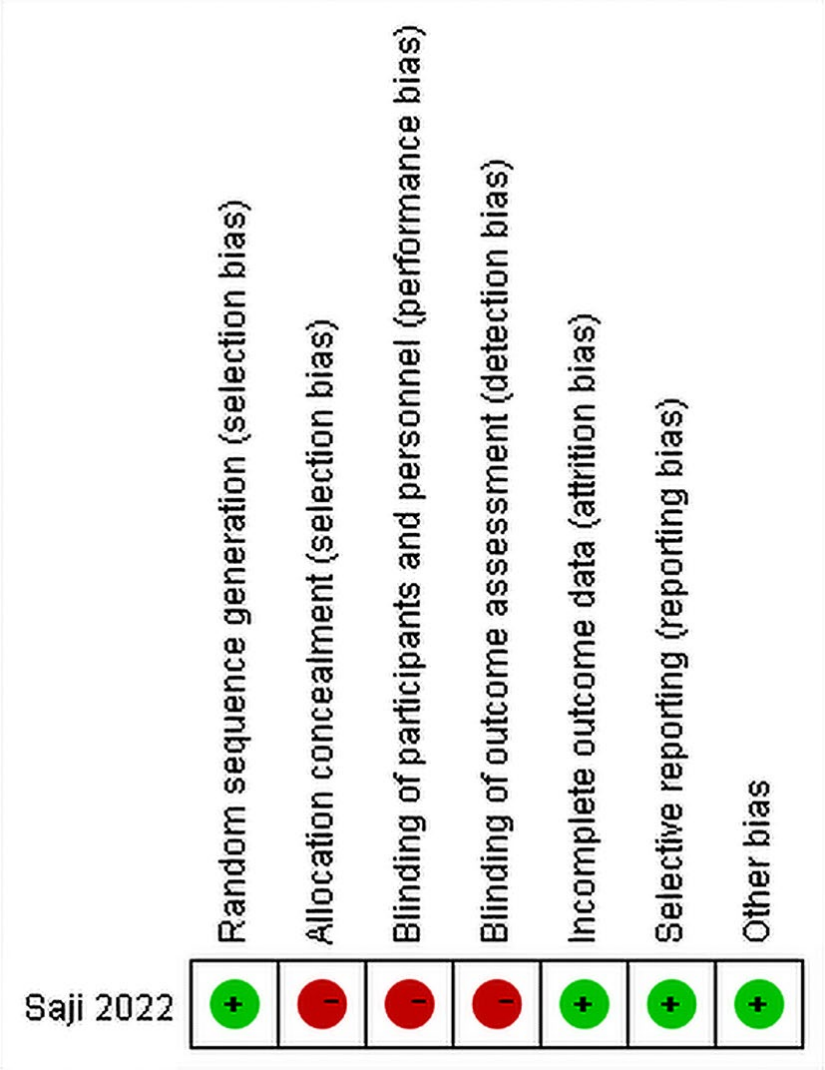
Risk of bias assessment of RCT

17 articles were included in the analysis. The quality of 16 cohort studies was assessed using the NOS, with 8 of these studies utilizing propensity score matching. The average NOS score for the cohort studies was 7.5. One randomized controlled study was evaluated using the Cochrane Collaboration tool, and it was determined to have a high quality(Fig. 2). A total of 4476 patients were included in the 17 studies, divided into two groups: the segmentectomy group (*n* = 2081) and the lobectomy group (*n* = 2395). All studies compared the survival outcomes between these two groups. The basic characteristics and quality evaluations of the studies are displayed in Table 1.

**Table 1:** The basic characteristics and quality evaluation of the study

First Author	Year	Country	Study Type	Propensity Score-Matched	Study Period	Stage	Segmentectomy	Lobectomy	Quailty Score
Yamashtia S	2011	Japan	Retrospective	No	2003-2011	I	90	124	7
Song CY	2018	Japan	Retrospective	Yes	2007-2016	IA	41	41	8
Kamigaichi A	2020	Japan	Retrospective	Yes	2007-2017	IA	37	37	7
Wen ZX	2020	China	Retrospective	Yes	2008-2018	IA	214	214	8
Zhong CX	2012	China	Retrospective	No	2006-2011	IA	40	40	8
Yamazaki	2021	Japan	Retrospective	Yes	2012-2019	IA	93	93	7
Kodama K	2016	Japan	Retrospective	Yes	1997-2010	IA	69	69	7
Ernest G	2020	USA	Retrospective	No	2003-2016	T1cN_0_M_0_	90	90	8
Warren and Faber	1994	USA	Retrospective	No	1980-1988	I	66	103	8
Kagimoto A	2021	Japan	Retrospective	No	2011-2020	I	108	186	8
Landreneau R-J	2014	USA	Retrospective	Yes	-	I	312	312	7
Hattori A	2015	Japan	Retrospective	No	2008-2013	T1_b_	31	123	7
Shapiro M	2009	Japan	Retrospective	No	2002-2008	I	31	113	7
Helminen O	2020	Finland	Retrospective	No	2007-2012	I	149	138	8
Hwang Y	2014	Korea	Retrospective	Yes	2005-2013	I	94	94	8
Labbouz S	2018	UK	Retrospective	Yes	2008-2016	I(T1a or T1_b_)	64	64	7
Saji H	2022	Japan	RCT	No	2009-2020	IA	552	556	-

### OS

**Fig. 3:**
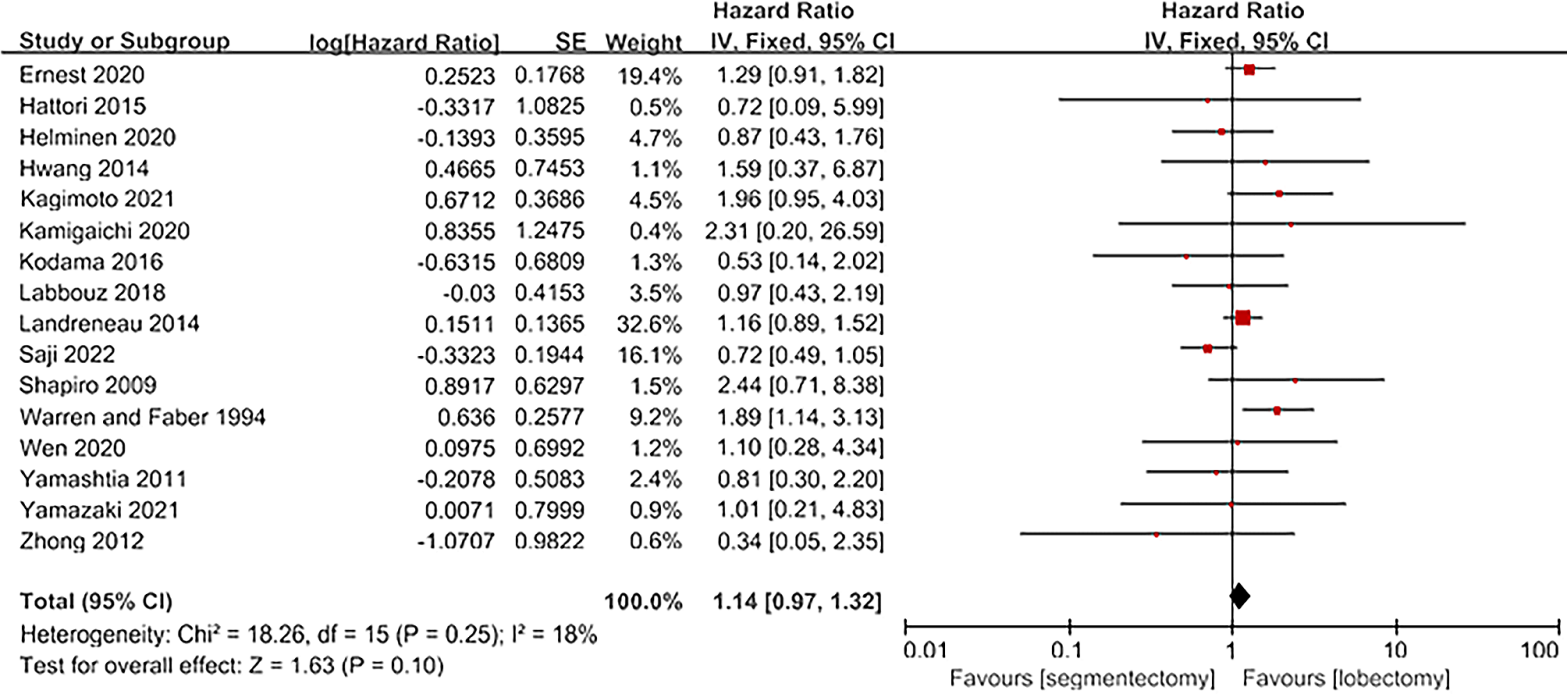
Forest plot for OS comparing segmentectomy to lobectomy

A total of 17 articles provided information on overall survival (OS) [4; 8; 9; 10; 11; 12; 13; 14; 15; 16; 17; 18; 19; 20; 21; 22]. These articles included a total of 4,478 patients, with 2081 in the segmentectomy group and 2397 in the lobectomy group. The heterogeneity of the literature was not significant (I^2^ = 18%, *P* = 0.25), and a fixed-effect model was used for analysis. The results indicate that there was no significant difference between the segmentectomy group and the lobectomy group for OS [HR = 1.14, 95%CI(0.97,1.32), *P* = 0.10; Fig. 3].

### DFS

**Fig. 4:**
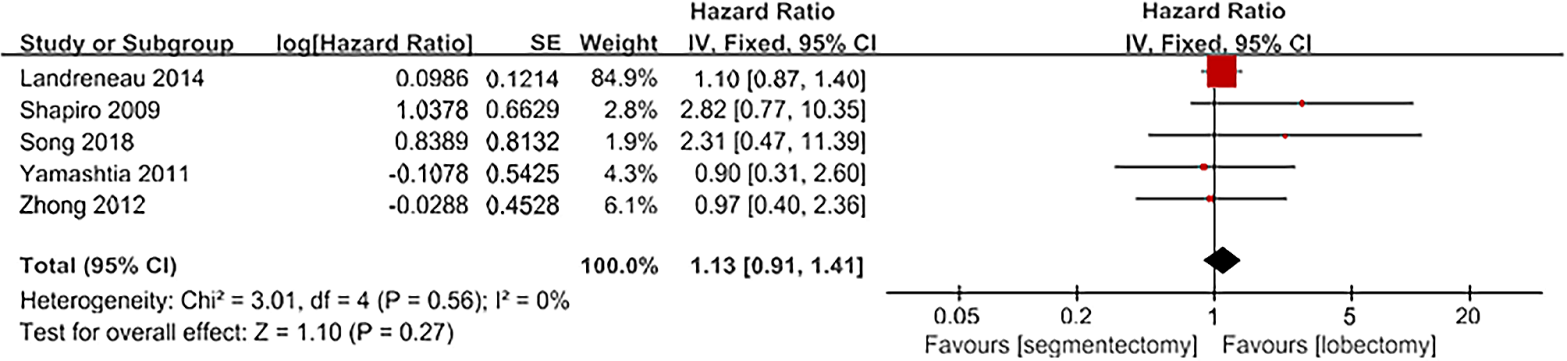
Forest plot for DFS comparing segmentectomy to lobectomy

A total of 5 articles reported on DFS [16; 19; 21; 22; 23]. These articles included a total of 1144 patients, with 514 in the segmentectomy group and 630 in the lobectomy group. The heterogeneity among the included literature was not significant (I^2^ = 0, *P* = 0.56) and a fixed-effect model was used for analysis. The results indicate that there was no significant difference between the segmentectomy group and the lobectomy group in terms of DFS. [HR = 1.13, 95%CI(0.91,1.41), *P* = 0.27; Fig. 4].

### RFS

**Fig. 5:**
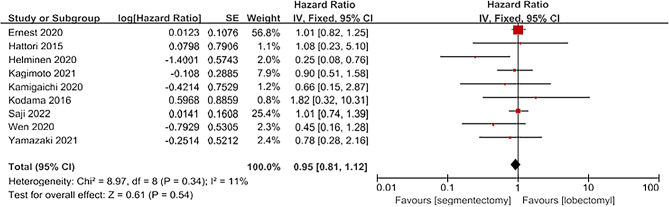
Forest plot for RFS comparing segmentectomy to lobectomy

A total of 9 articles reported on DFS [4; 8; 10; 11; 13; 14; 15; 17; 20]. The 9 articles included 2875 patients, including 1340 in the segmentectomy group and 1535 in the lobectomy group. The heterogeneity of the included literature was not significant(I^2^ = 11%, *P* = 0.34) and was analyzed using a fixed-effect model. The results show that for OS. There was no significant difference between segmentectomy group and lobectomy group[HR = 0.95, 95%CI(0.81,1.12), *P* = 0.54; Fig. 5].

### Propensity score-matched study results

**Fig. 6:**
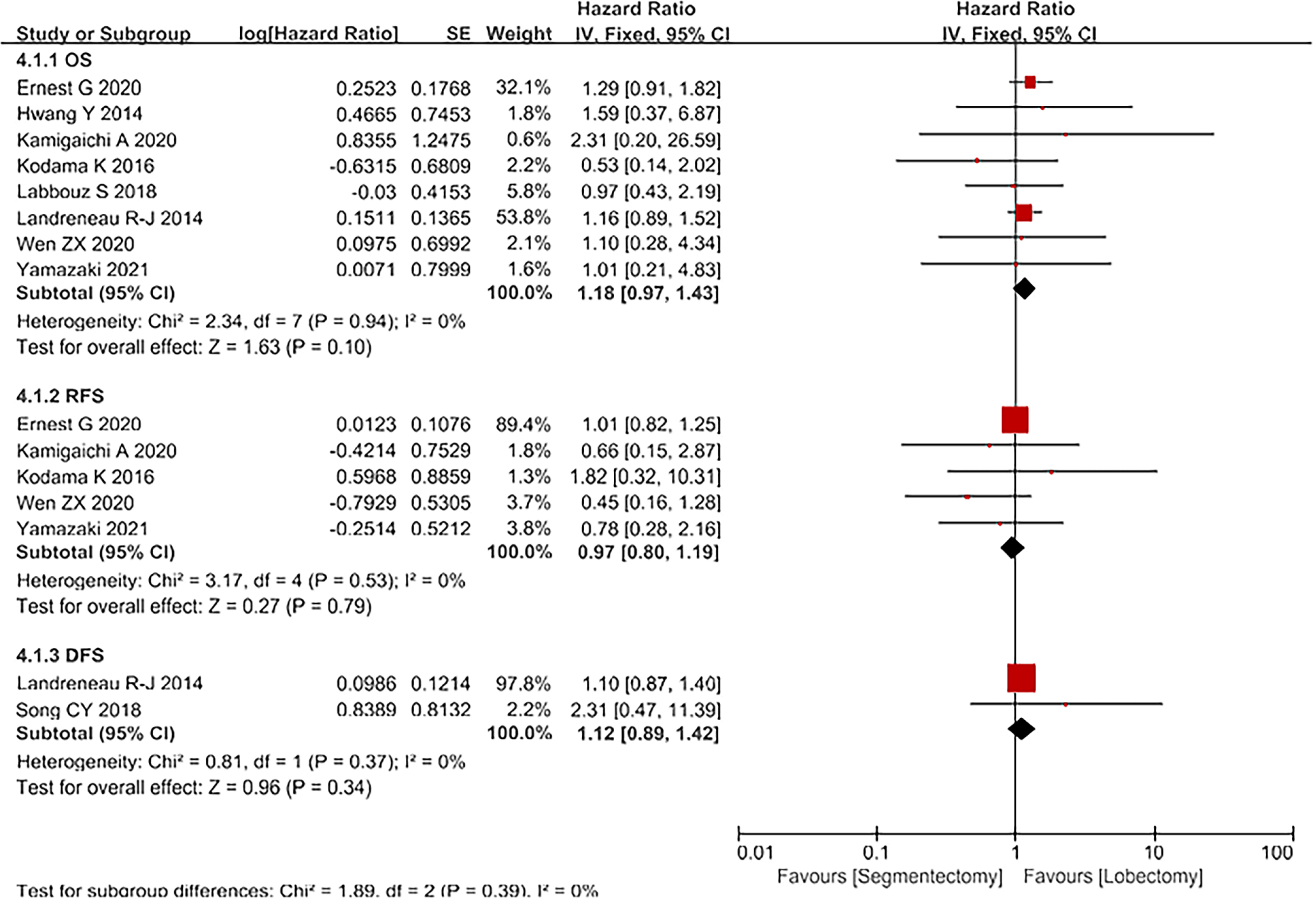
Propensity scoring studies’ forest plot for OS, DFS, RFS comparing segmentectomy to lobectomy

We separately analyzed the data which were designed as propensity scoring studies (Fig. 6). A total of 8 articles reported on OS [12; 14; 15; 16; 17; 18; 20; 24]. The heterogeneity of the included literature was not significant(I^2^ = 0%, *P* = 0.94) and was analyzed using a fixed-effect model. The results show that for OS. There was no significant difference between segmentectomy group and lobectomy group[HR = 1.18, 95%CI(0.97,1.43), *P* = 0.10]; A total of 5 articles reported on RFS [14; 15; 17; 20; 24]. The heterogeneity of the included literature was not significant(I^2^ = 11%, *P* = 0.34)and was analyzed using a fixed-effect model. The results show that for RFS. There was no significant difference between segmentectomy group and lobectomy group[HR = 0.97, 95%CI(0.80,1.19), *P* = 0.79]; A total of 2 articles reported on DFS [16; 23]. The heterogeneity of the included literature was not significant(I^2^ = 0%, *P* = 0.53) and was analyzed using a fixed-effect model. The results show that for RFS. There was no significant difference between segmentectomy group and lobectomy group[HR = 1.12, 95%CI(0.89,1.42), *P* = 0.34].

### Publication bias

Funnel chart shows clear symmetry, indicating no published bias (Suppl Fig. S1).

### Sensitive analysis

The sensitivity of each study was analyzed by sequentially removing one study at a time, and the results remained statistically significant. The postoperative OS combined hazard ratios and 95% confidence intervals (CI) ranged from 1.08 (0.92–1.27) to 1.24 (1.05–1.46) (Suppl Fig. S2). The RFS combined HRs and 95% CI ranged from 0.88 (0.69–1.12) to 0.98 (0.83–1.15) (Suppl Fig. S3). Lastly, the DFS combined HRs and 95% CI ranged from 1.14 (0.89–1.38) to 1.29 (0.73–2.27) (Suppl Fig. S4).

## Discussion

In 1995, the first randomized controlled trial comparing lobectomy and sublobectomy was conducted and the results showed that sublobectomy had a higher mortality rate and three times the local recurrence rate compared to lobectomy, making the latter the gold standard treatment for early non-small cell lung cancer (NSCLC) [3].

However, several studies have shown that segmentectomy and lobectomy result in similar overall survival rates and postoperative complications in patients with NSCLC less than 2 cm in size [25; 26; 27]. Additionally, both procedures yield better survival outcomes compared to non-surgical methods such as stereotactic ablative radiotherapy [28; 29]. The debate about whether segmentectomy should be performed for all patients with early NSCLC, rather than just those with limited cardiopulmonary function, continues.

To clarify this issue, we conducted a meta-analysis that included mostly medium to high quality retrospective studies. However, with only one randomized controlled trial (RCT) included, subgroup analysis was not possible.

Segmentectomy is now considered an alternative to lobectomy for early-stage lung cancer, particularly in cases of restricted lung function or comorbidities that make lobectomy challenging. Our meta-analysis found no significant difference in OS, DFS or RFS between segmentectomy and lobectomy, with low heterogeneity. This finding supports the notion that lung segmentectomy is a reasonable option for early-stage lung cancer patients. Other meta-analyses also support this conclusion. [30; 31; 32]. A meta-analysis by Cao et al [33] found no significant difference in OS and DFS between patients with early-stage NSCLC who intentionally opted for segmentectomy and those who underwent lobectomy. Conversely, patients with underlying conditions or limted cardiopulmonary function had significantly worse OS and DFS than patients with lobectomy. The results of a Phase III randomized controlled trial in Japan (JCOG0802 / WJOG4607L), published in April 2022, are encouraging [4]. The segmentectomy is a viable option. The 5-year survival rate for the segmentectomy group was higher than that of the lobectomy group, and the 5-year recurrence-free survival was similar. The National Cancer Institute (NCI) initiated its own Phase III randomized trial (CALGB 140,503) in 2008 to compare lobectomy and sublobectomy for the treatment of small peripheral NSCLC. The results of this trial have yet to be published.

Most trials demonstrating the superiority of lobectomy were not fully randomized and did not account for other variables that may affect survival. The lymph node yield with anatomical segmentectomy, a minimally invasive procedure, is notably lower compared to lobectomy. This disparity could stem from differences in the number of inter-segmental and intra-segmental lymph nodes extracted, as well as the preference for lymph node sampling over lymph node dissection during anatomical segmentectomy. The extent of lymph node dissection serves as a prognostic factor in the surgical management of early-stage non-small cell lung cancer [34], a finding that holds significance in segmentectomy as well. Evaluation of the SEER database by Qu et al [35] revealed that differences in survival between segmentectomy and lobectomy dissipated after stratifying patients based on the extent of lymph node dissection. Notably, this systematic lymph node dissection is not feasible in nonoperative modalities such as stereotactic ablative radiotherapy, highlighting a distinct advantage of surgical treatment. Tumor histology stands as another determinant of lung cancer prognosis.

In discussions concerning the role of segmentectomy, consideration of the consolidation-to-tumor ratio (C/T ratio) is warranted. Tumors with a higher C/T ratio have been demonstrated to exhibit greater invasiveness [36], and consequently, limited resection yields inferior overall survival (OS) and recurrence-free survival (RFS) compared to noninvasive tumors. Furthermore, a higher C/T ratio serves as an independent risk factor for local-regional RFS, rendering such tumors unsuitable for limited resection [37].

It should be noted that this review has some limitations, including a reliance on retrospective studies and potential bias that may influence the results. The results of the meta-analysis should be interpreted with caution due to the retrospective nature of most studies and heterogeneity between studies. Further evidence, particularly from prospective randomized controlled trials, is needed to definitively compare the survival outcomes of segmentectomy and lobectomy in treating early NSCLC.

## Conclusion

Our meta-analysis revealed that there was no significant difference in survival outcomes between segmentectomy and lobectomy for patients with stage I non-small cell lung cancer (NSCLC). It is worth noting that segmentectomy is also a viable treatment option for early stage NSCLC.

### Electronic supplementary material

Below is the link to the electronic supplementary material.

Supplementary Material 1

Supplementary Material 2

Supplementary Material 3

Supplementary Material 4

Supplementary Material 5

## Data Availability

No datasets were generated or analysed during the current study.
