# Supplementary figures and images for: Survival outcomes of segmentectomy and lobectomy for early stage non-small cell lung cancer: a systematic review and meta-analysis
Source: J Cardiothorac Surg. 2024 Jun 22;19:353. doi: 10.1186/s13019-024-02832-6 (PMC11193294; doi:10.1186/s13019-024-02832-6)

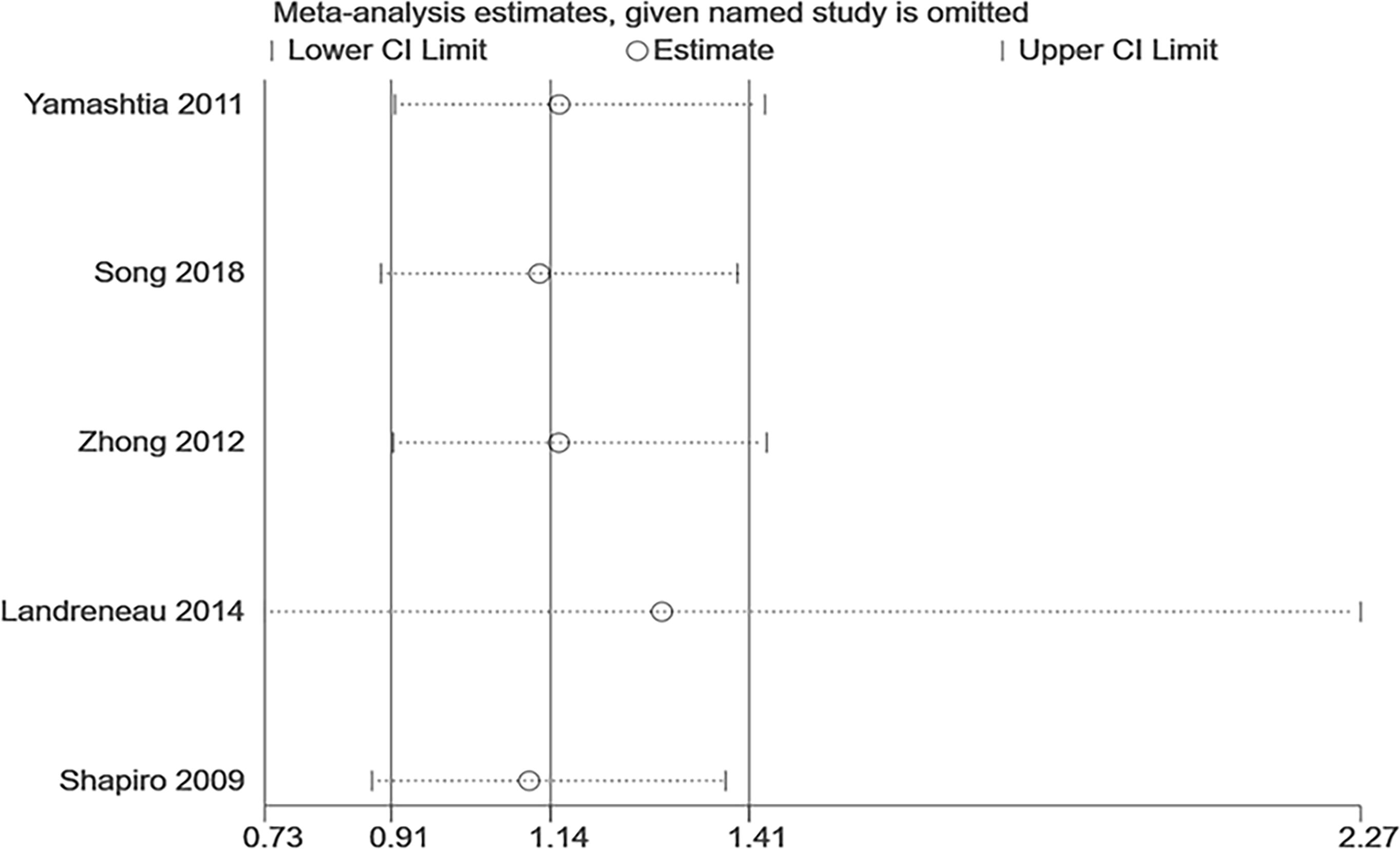

Supplement: Supplementary file 1 — Supplementary Material 1 [file 13019_2024_2832_MOESM1_ESM.png]

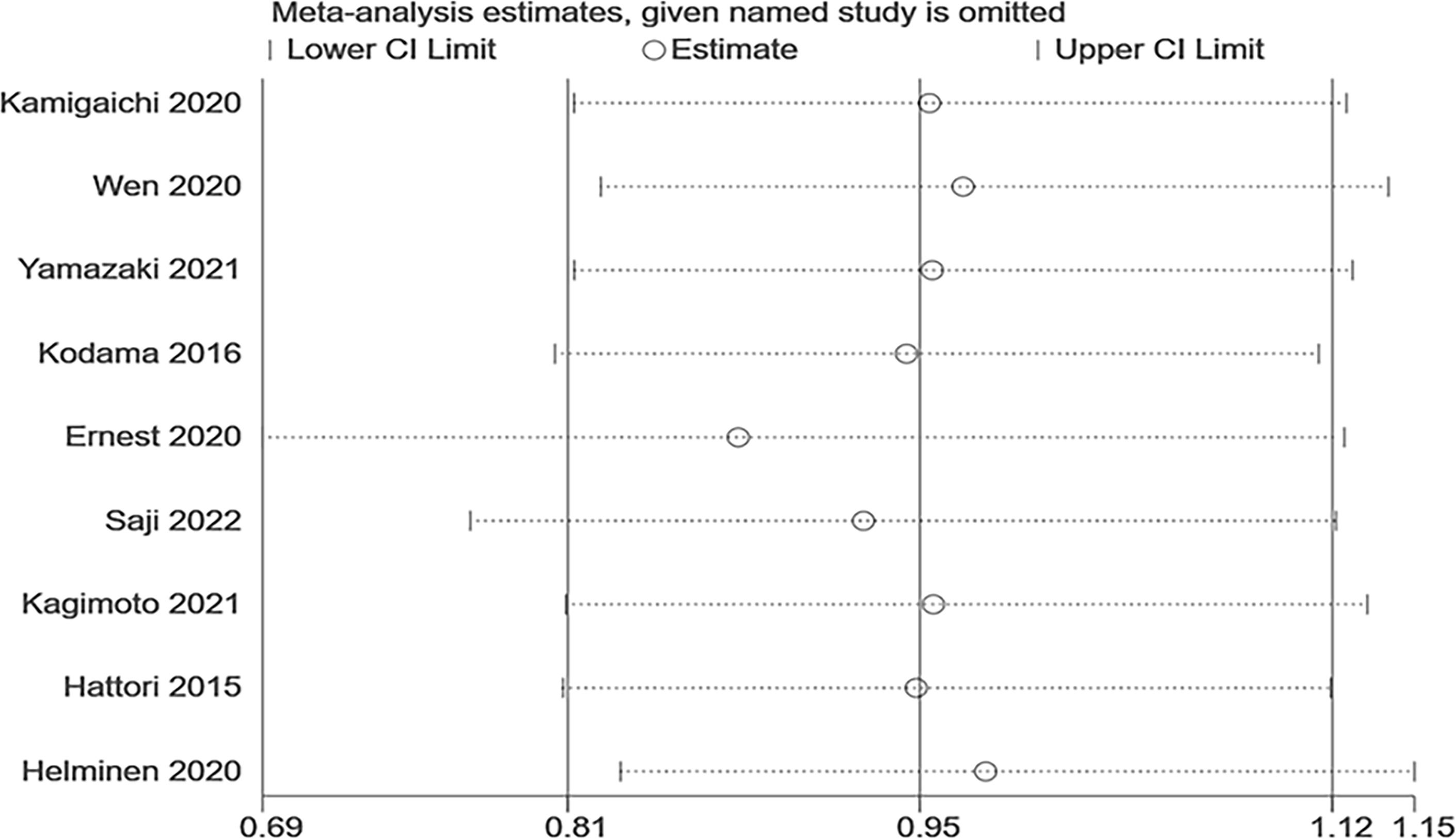

Supplement: Supplementary file 2 — Supplementary Material 2 [file 13019_2024_2832_MOESM2_ESM.png]

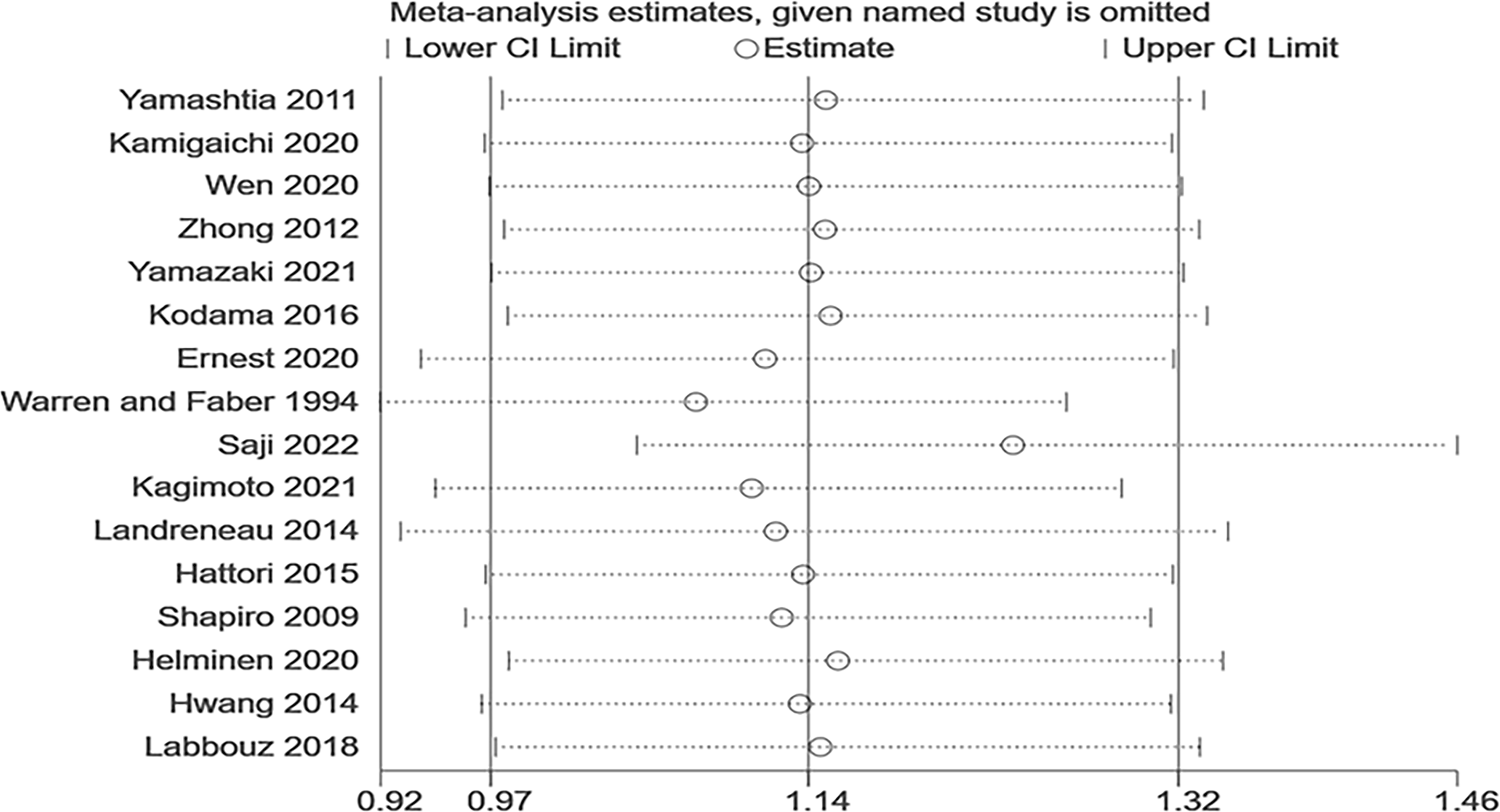

Supplement: Supplementary file 3 — Supplementary Material 3 [file 13019_2024_2832_MOESM3_ESM.png]

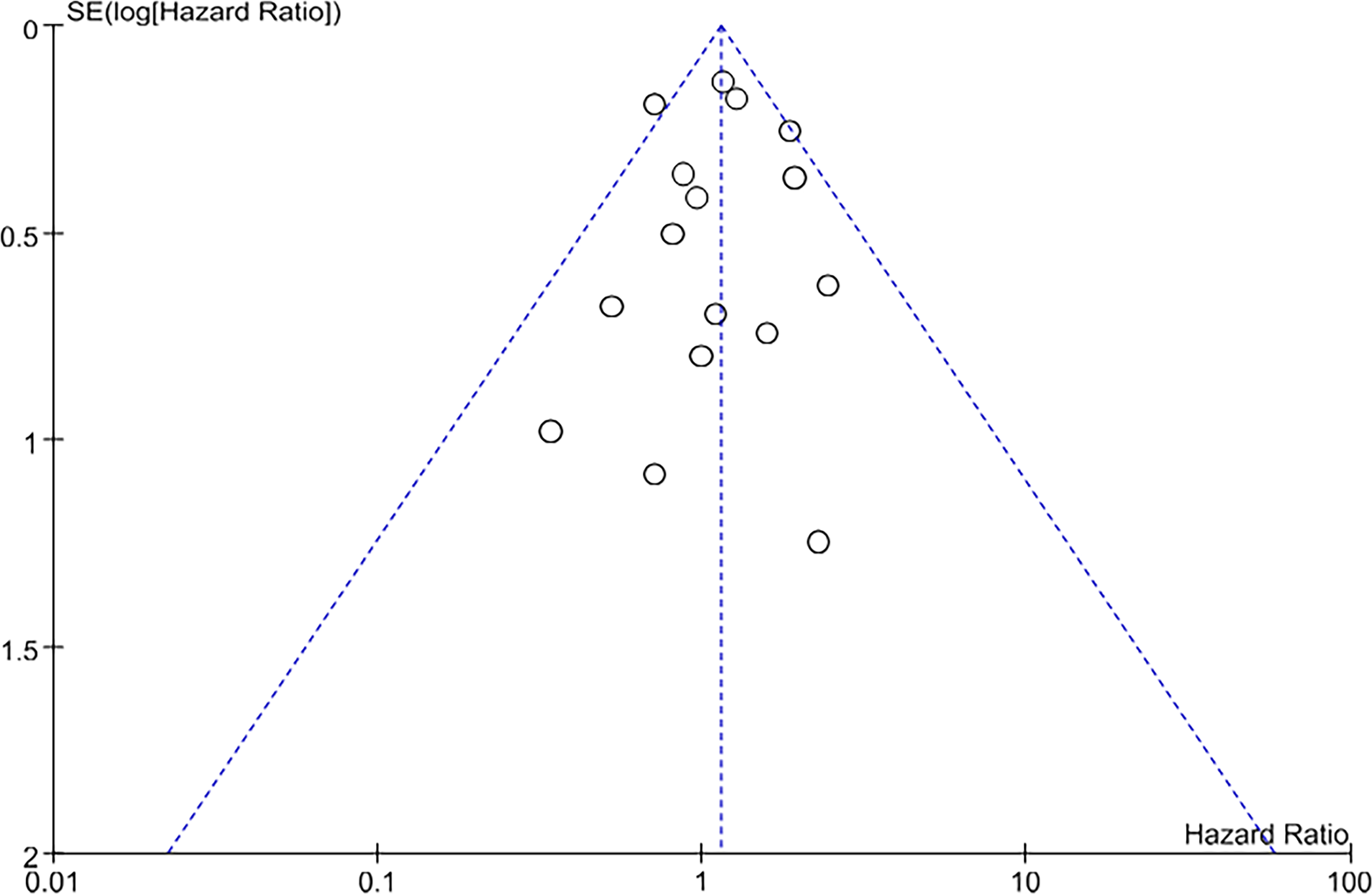

Supplement: Supplementary file 4 — Supplementary Material 4 [file 13019_2024_2832_MOESM4_ESM.png]
