# Supplementary material for: Survival outcomes of segmentectomy and lobectomy for early stage non-small cell lung cancer: a systematic review and meta-analysis
Source: J Cardiothorac Surg. 2024 Jun 22;19:353. doi: 10.1186/s13019-024-02832-6 (PMC11193294; doi:10.1186/s13019-024-02832-6)
Supplement: Supplementary file 5 — Supplementary Material 5 [file 13019_2024_2832_MOESM5_ESM.docx]

**The search terms were used as follows: ((Carcinoma, Non-Small-Cell Lung or Carcinoma, Non Small Cell Lung or Carcinomas, Non-Small-Cell Lung or Lung Carcinoma, Non-Small-Cell or Lung Carcinomas, Non-Small-Cell or Non-Small-Cell Lung Carcinomas or Non-Small-Cell Lung Carcinoma or Non Small Cell Lung Carcinoma or Carcinoma, Non-Small Cell Lung or Non-Small Cell Lung Carcinoma or Non-Small Cell Lung Cancer or Nonsmall Cell Lung Cancer) AND (Pneumonectomy or Pneumonectomies or Endoscopic Lung Volume Reduction or Partial Pneumonectomy or Partial Pneumonectomies or Pneumonectomy, Partial or Bronchoscopic Lung Volume Reduction or Lung Volume Reduction or Reduction, Lung Volume or Volume Reduction, Lung or Lung Volume Reduction Surgery or lobectomy)) AND (segmentectomy or segmental resection) AND (survival)**

**Search date up to July, 2022**

**Pubmed：549**

**Cochrane Library：51**

**Embase：1133**

**Total :1733**

**After duplication: 1320**
